# Parental Psychological Response to Prenatal Congenital Heart Defect Diagnosis

**DOI:** 10.3390/children12081095

**Published:** 2025-08-20

**Authors:** Cristina Tecar, Lacramioara Eliza Chiperi, Dafin Fior Muresanu

**Affiliations:** 1 RoNeuro Institute for Neurological Research and Diagnostic, 400364 Cluj-Napoca, Romania; cristina.pantelemon@umfcluj.ro (C.T.); dafin.muresanu@umfcluj.ro (D.F.M.); 2 Department of Neuroscience, Iuliu Hatieganu University of Medicine and Pharmacy, 400083 Cluj-Napoca, Romania; 3 Department of Pediatric Cardiology, Emergency Institute for Cardiovascular Diseases and Heart Transplant, 50 Gheorghe Marinescu Street, 540136 Targu Mures, Romania

**Keywords:** psychological, congenital heart disease, prenatal diagnosis, counseling, parent coping, review

## Abstract

Background: This systematic review aims to summarize the most recent data from the literature on the psychological aspects of parents of children prenatally diagnosed with congenital heart defects (CHDs). Methods: A comprehensive literature search was conducted to identify relevant studies on the psychological issues faced by parents of children prenatally diagnosed with CHD. Searches were performed in multiple scientific databases, including PubMed, Science direct, Embase, Scopus, Medline, Clarivate, to ensure the broad coverage of the literature. The search was limited to studies published up until February 2025. The search strategy included the following terms and combinations: “congenital heart defect” OR “CHD” AND “prenatal diagnosis” AND “psychological impact” OR “parental distress” OR “coping”. Results: Eighteen studies involving the 673 parents of fetuses diagnosed with congenital heart defects were included. Studies spanned four continents and employed both qualitative (n = 14) and quantitative (n = 4) designs. Key psychological outcomes reported were anxiety, depression, stress, post-traumatic stress, coping strategies, maternal–fetal attachment, and life satisfaction. Anxiety and depression were the most frequent issues, with maternal anxiety reaching 65% and depression up to 45.7%. Stress related to diagnostic uncertainty was common. While some parents used adaptive coping (social support, emotional regulation), others experienced maladaptive patterns such as avoidance. One study reported increased maternal–fetal attachment following prenatal CHD diagnosis. Predictors of psychological distress included time of diagnosis, parental gender, education level, social support, and severity of the defect. Recommended interventions included early psychological screening, empathetic communication, structured counseling, and long-term emotional support. Despite heterogeneity in design and moderate overall bias, findings highlight a consistent psychological burden among parents, underscoring the need for integrated psychosocial care following a prenatal CHD diagnosis. Conclusions: Parents whose children have been prenatally diagnosed with a congenital heart defect are at an increased risk for psychological distress. To improve the quality of care, a multidisciplinary team is needed to provide parents with the necessary information on diagnosis, interventions, and potential outcomes.

## 1. Introduction

### 1.1. Background Data

Congenital heart defects (CHDs) are a common type of major birth defect [1]. Worldwide, CHD prevalence is estimated at 8 per 1000 live births [2,3]. It is known that, thanks to the progress made in the last three decades in the medical and surgical treatment of CHDs, the survival rate of these children has reached 85–90%. A study by Oster et al. showed that 1-year survival of children with critical CHD improved from 67% for the 1979–1993 era to 85% for the 1994–2005 era [4]. Although much progress has been made in the management of this condition, subsequent outcomes in terms of performance and physical ability vary due to the variety of CHD. CHD can range from mild CHD, in which the patient shows no obvious symptoms and the malformation may be discovered by chance in adulthood to severe CHD that can cause premature death [5]. Thanks to the advances in fetal heart and cardiovascular system examination, over the last two decades, the accurate prenatal diagnosis of all forms of CHD is possible [5]. Nowadays, the aim is to understand the fetus as a patient in its own right, knowing that the fetal circulation is different from the postnatal circulation, the structural heart malformation may progress in utero, the cardiac function and the stability of the cardiovascular system play an important role in fetal development [1].

Another important aspect to consider is how prenatal maternal stress—especially when triggered by receiving a diagnosis of a fetal congenital heart defect (CHD)—can affect both fetal development and long-term child outcomes [6]. This stress may arise from emotional shock, uncertainty, or anticipatory grief following diagnosis. Research over the past two decades has shown that maternal stress during pregnancy is associated with a higher risk of emotional, behavioral, and cognitive problems in children, such as anxiety, depression, attention-deficit/hyperactivity disorder (ADHD), and autism spectrum disorders [7,8,9,10]. These findings are particularly relevant when considering the potential bidirectional impact: not only does the diagnosis affect the parent psychologically, but the maternal psychological state may, in turn, influence fetal neurodevelopment. Thus, understanding and addressing parental psychological responses following a prenatal CHD diagnosis is essential for both parental well-being and optimal child development.

Animal studies have shown that the activity of the stress-responsive-pituitary-adrenal (HPA) axis and its end-product cortisol are involved in both the effect of stress on the mother and the fetus [11]. Studies have also shown that depression in fathers in the postnatal period is associated with psychiatric disorders in children 7 years later, independent of maternal postnatal depression [12].

The prenatal diagnosis of CHD is an unexpected event for the parents that can cause shock, guilt, grief and anger [13]. These parents are at an increased risk for developing anxiety, depression and post-traumatic stress [14,15,16]. A systematic review by King et al. [17] showed that up to 30% of parents of children with critical CHD had post-traumatic stress symptoms, more than 80% of them had clinical symptoms of trauma, 25% to 50% of them had symptoms of depression and/or anxiety, and 30–80% of them reported severe psychological distress, especially immediately after the child’s surgery [17]. Johnson et al. identify five stages in the parents’ response to their child’s diagnosis: expectations for ultrasonography, discovery of the malformation, shock, uncertainty and decision making, and adjustment to the diagnosis [18]. Fonseca et al. showed that 40% of parents whose children were diagnosed with a congenital malformation had a CHD had higher levels of stress than parents of healthy children, even though they had the same quality of life. Another aspect highlighted in this study was that learning the diagnosis prenatally was associated with a higher quality of life for mothers compared to those who learned the diagnosis after the birth of the child [19]. Bevilacqua et al. [20] found no difference between the level of stress and depression in both mothers and fathers who were diagnosed with congenital heart defects prenatally or postnatally. Mothers whose babies were diagnosed prenatally were more likely to be depressed, while those who were diagnosed postnatally were more likely to be stressed. Also, in this study, the levels of stress and depression reported by parents two weeks after hospitalization of the child in the first 3 months of life were higher in the mother than in the father [20].

There are many studies conducted that highlight the psychological impact on parents of children with severe conditions such as cancer, while there are few studies that show the impact of a congenital heart malformation diagnosis on the mental health and wellbeing of parents. Most studies conducted on the parents of children with CHD focus on their overall quality of life [21]. Once the diagnosis of congenital heart malformation is communicated, it is important to support parents in using coping mechanisms based on their own resources (emotional, cognitive and economic) [6]. It is important to provide specialized support to parents in understanding the information provided regarding the diagnosis and decision making [22,23,24]. Increased levels of acceptance and correct management have been associated with lower levels of anxiety, depression, and post-traumatic stress [25].

### 1.2. Aim

This review aims to summarize the most recent data from the literature on the psychological aspects among parents of children prenatally diagnosed with CHD. It also emphasizes the early importance of adequate psychological support provided to the parents of these children by a multidisciplinary team.

## 2. Materials and Methods

### 2.1. Literature Search

In this systematic review, a comprehensive literature search was conducted to identify relevant studies on the psychological issues faced by parents of children diagnosed prenatally with CHD. Searches were performed in multiple scientific databases, including PubMed, Science direct, Embase, Scopus, Medline, Clarivate, to ensure the broad coverage of the literature. The literature search covered all eligible studies published from database inception through July 2025, with no language restrictions and was conducted using PRISMA guidelines [26].

### 2.2. Search Strategy

The search strategy included the following terms and combinations: “congenital heart defect” OR “CHD” AND “prenatal diagnosis” AND “psychological impact” OR “parental distress” OR “coping”. Boolean operators and MeSH terms were used when available. No date or language restrictions were applied. Filters were applied to include only human studies. The complete search strategy for each database is provided in Supplementary File S1.

### 2.3. Inclusion and Exclusion Criteria

Studies were included if they provided empirical data (qualitative or quantitative) on the psychological effects of prenatal diagnosis of congenital heart defects on parents. This included studies that assessed specific psychological constructs such as anxiety, depression, stress, coping mechanisms, or post-traumatic symptoms using validated tools or structured interviews.

We excluded studies that only made general statements about parental emotional responses without supporting data (e.g., “parents were distressed” without scales or qualitative coding), studies focusing solely on medical decision making or pregnancy outcomes without exploring the psychological component, and articles describing institutional protocols or genetic counseling frameworks without parental outcome data. Additionally, protocols were classified as inadequate if they lacked clarity in population selection, failed to specify psychological endpoints, or used non-validated instruments.

### 2.4. Study Selection and Assessment

The studies identified were independently screened and selected by two reviewers to ensure objectivity in the selection process. Discrepancies were resolved through discussion. However, no formal inter-rater reliability index (e.g., Cohen’s Kappa) was calculated. This lack of a quantified inter-rater agreement represents a methodological limitation of the study and is acknowledged as such in the limitations section. After the initial selection, the studies were assessed for methodological quality using ROBINS-I, a Cochrane Risk of Bias Tool.

### 2.5. Data Extraction

Relevant data from each selected study were extracted using a standardized data extraction form. The information extracted included (1) Author, year; (2) Study design; (3) The geographic area in which the study was conducted; (4) Number of parents included; (5) Type of tools used to assess psychological issues; (6) Specific domain of research related to parental psychological issues; (7) Follow-up period; (8) Predictors or modifiers of psychological distress; (9) Main outcomes related to the psychological impact on parents; and (10) Recommended interventions for reduction in psychological distress.

Additional data items collected included study setting, parental demographics (e.g., age, gender), timing of CHD diagnosis (prenatal vs. postnatal), severity of the condition, and any reported interventions. Where information was unclear, assumptions were made based on context or study design.

### 2.6. Data Synthesis

Regarding data analysis, a narrative synthesis of the results from the included studies was performed, considering the diversity of the methods used and the varied nature of the data.

### 2.7. Study Quality and Risk of Bias Assessment

Given the mixed-methods nature of the included studies—both qualitative and quantitative designs—different tools were used for assessing methodological quality. For qualitative studies, the ROBINS-I instrument was used, and for quantitative studies, CASP (Critical Appraisal Skills Programme) was applied. This evaluation was used to discuss potential sources of bias and to interpret the results accordingly.

No formal statistical methods (e.g., funnel plots or Egger’s test) were applied to assess reporting bias due to the predominance of qualitative studies. However, potential publication bias was considered based on the lack of studies with null or negative findings and an overrepresentation of small, single-center qualitative studies.

A formal GRADE assessment of the certainty of evidence was not performed due to the heterogeneity and qualitative nature of most included studies. Instead, overall confidence in the findings was discussed narratively, considering study design, consistency of results, and risk of bias assessments.

## 3. Results

### 3.1. Study Selection

The initial literature search identified a total of 1394 records across databases: PubMed (412), Embase (237), Scopus (303), ScienceDirect (195), Clarivate (147), and Medline (100). After removing 38 duplicates, 1356 unique records remained for screening.

Title screening excluded 1280 articles, primarily due to the following: (1) studies not addressing congenital heart disease (n = 721), (2) studies unrelated to psychological or parental outcomes (n = 365), and (3) commentaries or editorials (n = 194).

Out of 76 abstracts assessed for eligibility, 27 were excluded due to(1) focus on postnatal-only diagnoses (n = 14), (2) absence of psychological outcome data (n = 9), and (3) non-parental perspectives (n = 4).

Although 49 articles met abstract eligibility, only 25 were retrieved in full text due to (1) unavailability through institutional access (n = 16), (2) duplicate reporting (n = 5), and (3) language restrictions with no translation available (n = 3).

**Figure 1:**
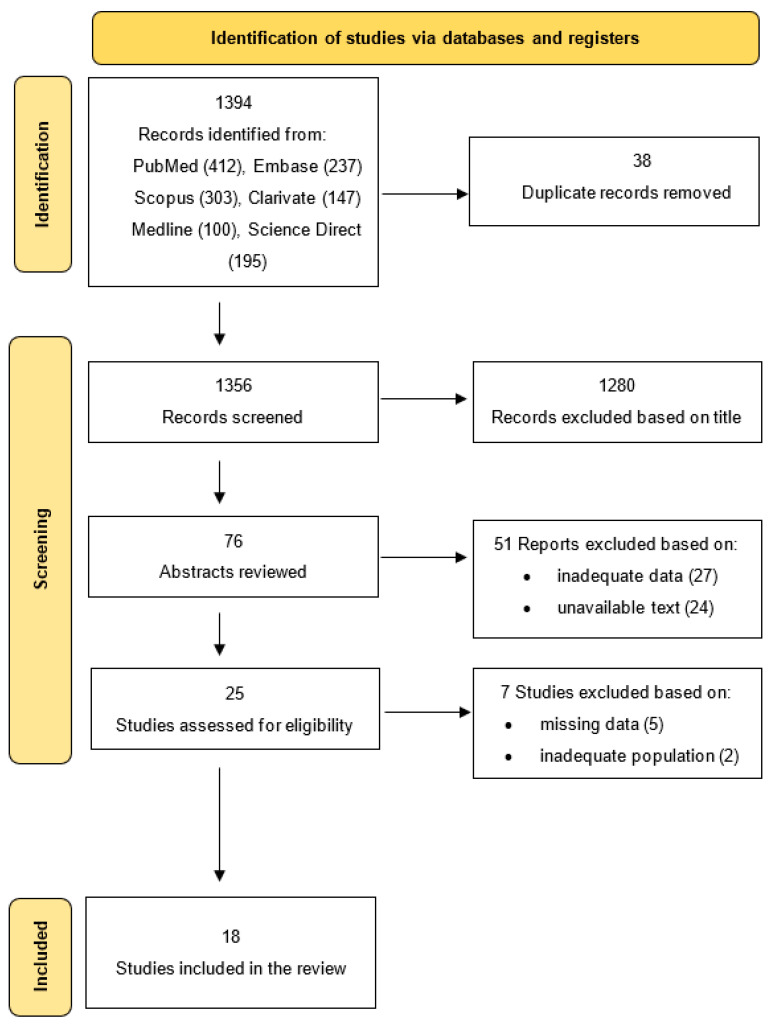
PRISMA flow diagram for new systematic review: selection process of included studies.

After full-text review, 7 studies were excluded for (1) inadequate population (n = 2) and (2) lack of psychological outcome measures (n = 5). Eighteen studies met all inclusion criteria and were included in the final synthesis. The revised PRISMA flow diagram is shown in Figure 1, and excluded full-text studies are detailed in Supplementary File S2.

### 3.2. Study Characteristics

The characteristics of the studies included in this review are summarized in Table 1. In total, 18 studies were included, comprising 673 participants who were parents to unborn fetuses with CHD.

The included studies were published over a period of 18 years; the earliest study was published in 2007, and the most recent study was from 2025. This 18-year span reflects a growing body of research on parental psychological responses to a prenatal CHD diagnosis.

The studies varied in design. Among them, 14 studies employed qualitative methods, using interviews or thematic analysis to explore parental experiences, perceptions, or coping strategies. Four studies used quantitative designs [23,28,33,37], typically employing validated psychometric scales like Edinburgh Postnatal Depression Scale (EPDS), Depression Anxiety Stress Scales (DASS) and State-Trait Anxiety Inventory (STAI) to assess symptoms of depression, anxiety, or stress in parents [23,28,33,37]. No mixed-methods studies were identified. This distinction is critical in evaluating the appropriateness of quality assessment tools and synthesizing evidence in a meaningful manner. A full breakdown of study design classification is available in Supplementary File S3.

The studies were conducted in various geographical locations spanning over four continents, including Europe (Sweeden, Italy, Portugal, Germany), North America (USA), South America (Brazil), Asia (Republic of Korea), and including 20 medical tertiary centers.

The participants in the included studies were the parents of fetuses diagnosed with a CHD (n = 673 parents), namely mothers and fathers with various demographic characteristics, as shown in Table 1.

The studies used interviews, live or via telephone, in which different questionnaires or scales like the Maternal–Fetal Attachment Scale [41], Edinburgh Postnatal Depression Scale [42], Perceived Stress Scale [42], Spielberger State-Trait Anxiety Inventory [43], Hospital Anxiety and Depression Scale [44], Dyadic Adjustment Scale [45], COPE Inventory [46], General Health Questionnaire-30 [47], Beck Depression Inventory—Second Edition [48], Health Survey-36 [49], Impact of Events Scale-Revised [50] or Brief Symptom Inventory [51] were completed.

Several studies incorporated follow-up periods to assess the psychological and emotional impact of a prenatal CHD diagnosis on parents. Key findings included short-term follow-ups (weeks to months after diagnosis or birth) in three studies [34,35,37], medium-term follow-ups (several months to a year) in three studies [28,29,30], and long-term follow-ups (more than a year after birth) in two studies [31,33].

### 3.3. Study Domain of Research and Outcomes

Several key psychological issues among parents of children diagnosed prenatally with CHD analyzed in included studies were anxiety, depression, stress, post-traumatic stress, adaptative process and coping mechanisms, attachment, life satisfaction, and mental health/wellbeing. The results of the systematic review include the following data:

**Anxiety:** Many studies (n = 6) reported that parents, particularly mothers, experienced high levels of anxiety following the prenatal diagnosis [25,32,33,34,37,40]. Reported anxiety levels varied, with some studies showing rates as high as 65% among mothers [32].

**Depression:** Depression was another common psychological issue, with six studies reporting elevated depressive symptoms among parents [20,25,27,29,32,34]. Depression rates varied across studies, with some reporting up to 45.7% of mothers experiencing depressive symptoms [20].

**Stress:** Parents also reported experiencing significant stress, particularly related to concerns about their child’s health and the impact of the diagnosis on family life. Five studies highlighted the emotional and psychological burden caused by the uncertainty of the child’s prognosis [30,33,36,37,39].

**Post-traumatic stress:** One study identified post-traumatic stress symptoms in parents, with some reporting intrusive thoughts and flashbacks related to the prenatal diagnosis and the possibility of severe outcomes for their child [25].

**Coping mechanisms:** Some studies (n = 2) explored how parents coped with the prenatal diagnosis. Common coping strategies included seeking social support, focusing on the positive aspects of the pregnancy, and engaging in health-promoting behaviors [25,31]. However, some parents were reported to struggle with maladaptive coping mechanisms, such as avoidance and denial [25,31].

**Attachment:** One study suggested that fetal CHD diagnosis increases maternal–fetal attachment levels and that fetal diagnosis should be offered to all mothers [38].

**Life satisfaction and mental health/wellbeing:** Two studies concentrated on the quality of life of the parents. They concluded that parents with a prenatal CHD diagnosis experience lower life satisfaction [28,34].

**Adaptative processes:** Several studies (n = 4) explored how parents adapt emotionally and psychologically after receiving a prenatal diagnosis of CHD [28,31,33,35]. Parents adapt to a prenatal CHD diagnosis through emotional regulation, information processing, and support systems. Mothers and fathers cope differently, requiring personalized support strategies.

### 3.4. Predictors or Modifiers of Psychological Distress

Several studies identified factors influencing psychological distress in parents after a prenatal CHD diagnosis. These factors can either increase (risk factors) or decrease (protective factors) distress levels. The most influential factor reported by multiple studies was time of prenatal diagnosis.

**Parental gender** was associated with psychological distress in multiple studies. Mothers were more likely than fathers to report depressive symptoms and stress [20,30,39]. In particular, Bevilacqua et al. [20] reported that 45.7% of mothers experienced depression compared to 20% of fathers, while 81.8% of mothers reported stress compared to 60.6% of fathers.

**Partner satisfaction** was identified as a modifying factor in a study by Rychik et al. [25], where lower partner satisfaction was correlated with higher levels of maternal depression and anxiety.

**Parental demographics**, including level of education, profession (especially healthcare or social work), first-time motherhood, and medically assisted pregnancy, were found to influence psychological outcomes [29].

**Severity of the CHD** also played a role. Brosig et al. [39] found that the severity of the lesion at diagnosis was directly related to parental distress scores.

**Time of diagnosis** emerged as the most influential factor, cited by multiple studies [27,31,34,36,38,40]. Prenatal diagnosis was associated with both protective and adverse psychological effects, depending on the context and available support.

**Uncertainty** was frequently cited as a major source of distress. Harris et al. [33] emphasized that uncertainty surrounding the diagnosis, prognosis, and next steps was a central theme in parental interviews.

**Social support** was another important protective factor. Lack of support systems, particularly for mothers, was associated with higher distress levels [30,35]. In contrast, stronger social and emotional support was linked to better psychological outcomes [28].

### 3.5. Recommended Interventions for Reducing Psychological Distress

The studies highlighted several evidence-based interventions to help parents manage psychological distress after a prenatal CHD diagnosis. These interventions focused on emotional support, communication, and coping strategies.

**Early and clear medical communication** was consistently recommended. Parents valued honest and detailed explanations from medical professionals, both verbal and written, tailored to their level of understanding. This was emphasized in studies by Carlsson et al. [22,23], Bratt et al. [37], and Harris et al. [33].

**Psychological support and counseling** were proposed in several studies. Mangin-Heimos et al. [30] recommended early and repeated psychological screening for both mothers and fathers. Bevilacqua et al. [20] emphasized the need for counseling throughout pregnancy to support parents as they adjust emotionally to the diagnosis.

**Social and peer support systems** were also identified as helpful. Studies by McKechnie et al. [28] and Demianczyk et al. [31] noted that collaborative efforts between healthcare providers and parents, including peer connections, helped build coping skills and reduced emotional burden.

**Long-term follow-up and postnatal support** were emphasized as crucial elements in several studies [28,30,31,37]. Continued contact with specialist nurses, multidisciplinary counseling teams, and access to online or community resources were all suggested to improve parental adjustment and reduce anxiety, depression, and stress.

**Tailored interventions** based on parental characteristics (e.g., gender, prior mental health, support networks) were advocated in studies by Erbas et al. [29] and Wu et al. [32], indicating that individualized support plans might enhance psychological resilience.

### 3.6. Study Quality and Risk of Bias

Of the 18 studies included in this review, 11 were classified as quantitative and 7 as qualitative, as can be seen in Table 1. Risk of bias was assessed separately using ROBINS-I for quantitative studies and the CASP checklist for qualitative studies, to better align with methodological standards.

Among the quantitative studies, two were rated as low risk of bias [29,30], eight were considered to have moderate risk [20,25,27,32,36,38,39,40], and one study was classified as high risk of bias [34], as can be seen in Table 2.

For the qualitative studies, based on the CASP assessment, five were considered to have moderate quality [22,23,28,31,35], and three studies were rated as low-to-moderate quality [33,37]. No qualitative study received a high CASP score or a low risk of bias rating, as can be seen in Table 3.

The most frequent sources of bias in qualitative studies were related to small sample size, lack of methodological transparency, and exclusive reliance on self-reported outcomes without triangulation. In quantitative studies, the main concerns included uncontrolled confounding variables, selection bias, and missing outcome data, particularly in studies with retrospective or self-selected cohorts.

**Bias due to confounding** was present in studies that did not account for variables such as socioeconomic status or pre-existing mental health conditions, which could influence psychological outcomes. High risk of confounding was identified in Bevilacqua [20], Rychik [25], and Bratt [34], while studies such as Erbas [29] and Mangin-Heimos [30] were considered at low risk due to the inclusion of psychological screening and demographic controls.

**Bias in participant selection** was noted in studies with small, self-selected samples, such as Bratt [37], Im [35], and Brosig [39]. In contrast, Ruschel [38] and Mangin-Heimos [30] included larger, more diverse samples, reducing this risk.

**Bias in the classification of interventions** was moderate in studies like Pinto [36] and Vieira [27], where prenatal versus postnatal diagnosis groups were defined but not always stratified by severity.

**Bias due to deviations from intended interventions** was low across all studies, as most were observational and did not implement active interventions.

**Bias due to missing data** was high in small-sample qualitative studies such as those of Bratt [34] and Im [35], where loss to follow-up or incomplete datasets were more likely. Moderate risk was found in McKechnie [28] and Demianczyk [31], which reported follow-up assessments but lacked full retention statistics.

**Bias in outcome measurement** was high in studies that solely relied on self-reported data, such as those of Harris [33] and Carlsson [23], which may have introduced subjective bias. In contrast, Erbas [29] and Ruschel [38] used validated instruments, resulting in moderate risk.

**Bias in the selection of reported results** was moderate overall. Studies like those of Demianczyk [31] and Pinto [36] may have selectively emphasized certain psychological dimensions (e.g., coping or stress), while Erbas [29] and Mangin-Heimos [30] provided more comprehensive outcome reporting.

### 3.7. Heterogeneity and Publication Bias

Substantial heterogeneity was observed across studies, primarily due to differences in study design, measurement tools, and participant characteristics. A sensitivity analysis was performed to explore the potential sources of heterogeneity. Two critical aspects to consider are heterogeneity and publication bias. Sources of heterogeneity in the included studies were represented by (a) study designs and methods; (b) study populations and sample sizes; (c) psychological outcomes measured. Indicators of potential publication bias in the included studies were (a) lack of studies with negative or null findings; (b) overrepresentation of qualitative and small-sample studies; and (c) geographic and language bias.

## 4. Discussion

This systematic review synthesizes the most recent data from the literature on the psychological stress experienced by the parents of children prenatally diagnosed with a congenital heart defect. The prenatal diagnosis of a medical condition creates a window of time (from weeks to months) before treatment implementation. In this time frame, there is an opportunity to educate and counsel the parents about the diagnosis and also to develop an optimal strategy for delivery and postnatal care [25]. Despite these benefits, antenatal diagnosis can create a prolonged period of stress for parents. In the next section, we present various aspects/dimensions of psychological distress experienced by the parents of children with congenital heart defects.

### 4.1. Anxiety

The prenatal diagnosis of a congenital heart defect is a stressful event that can affect the mother’s mood and increase anxiety. A study by Sklansky et al. [40] showed that, when fetal echocardiography diagnosed a congenital heart defect, the mother’s anxiety increased and they were less happy about being pregnant. However, mothers who had given birth to a baby with a congenital heart defect said that, following fetal echocardiography in pregnancy, they felt less responsible for the baby’s condition, and their relationship with the baby’s father improved after the congenital heart defect diagnosis [40]. In terms of the timing of diagnosis, Pinto et al. showed that contrary to previous reports, the parents of children diagnosed prenatally with a congenital heart defect had lower levels of global stress and anxiety than those diagnosed postnatally, after adjusting for severity. This may be due to improvements in prenatal support and the multidisciplinary services provided to families with a prenatal diagnosis of congenital heart malformation [36]. It is known that maternal stress during pregnancy has an adverse impact on pregnancy outcome. Studies have shown that anxiety increases the risk of obstetric complications (i.e., preeclampsia, miscarriage, premature birth) and is also related to altered fetal programming and emotional, cognitive and behavioral consequences in the infant and later in the adult [52,53,54,55]. Wu et al. have shown that psychological distress in pregnant women with children with congenital heart defects is associated with impaired fetal cerebellar and hippocampal development [32]. In a study by Bratt et al., parents whose children were diagnosed with a congenital heart defect reported increased levels of anxiety and depression compared to the parents of healthy children [37].

### 4.2. Depression

When a pregnant woman faces unexpected events during pregnancy she may experience emotional fragility and depression. It is known that mental health problems are the most common complication of pregnancy and affect up to 22% of women prenatally or in the first year after giving birth [56]. This percentage almost doubles in high-risk pregnancies, including women whose babies are diagnosed with congenital heart defects [25,39]. In a study by Vieira et al., they showed that the prenatal diagnosis of a critical congenital heart defect was associated with lower levels of depressive symptoms than postnatal diagnosis. Mothers of hospitalized infants experience higher levels of depressive symptoms, whereas those who have had time to familiarize themselves with their child’s diagnosis tend to be more optimistic [27].

### 4.3. Stress

Parents who receive a prenatal diagnosis of congenital heart malformation may experience greater short- or long-term stress than those who receive a postnatal diagnosis [5]. However, prenatal diagnosis is preferable as it has additional benefits in terms of emotional processing, education, and preparation for the birth [57,58]. Indeed, some parents who receive such a diagnosis may experience stress, anxiety, and depression a few months after birth [37,39,59]. When faced with the uncertainty of a prenatal diagnosis of congenital heart defect, parents seek information to reduce uncertainty and a strategy to cope with it [22,37,60]. The stress experienced by the parents of these children may have a greater effect on the quality of life of these children than the condition itself [61,62,63]. A study by Harris et al. identified aspects of counseling by the pediatric cardiologist that parents of children prenatally diagnosed with a congenital heart defect valued most: verbal and written communication in lay language about the diagnosis, resources for next steps, and the patience and kindness with which they were approached. Parents appreciated that they were presented with a plan for what would happen from the time of diagnosis into the distant future [33]. Asplin et al. showed that mothers want to be informed in the best- and worst-case scenarios [64]. This approach has been adopted for patients requiring palliative care to cope with uncertainty [65].

### 4.4. Post-Traumatic Stress

Post-traumatic stress is commonly seen in mothers after the prenatal diagnosis of congenital heart defects. Rychik et al. showed in a study that of, all mothers whose children were diagnosed with a congenital heart defect, 39% experienced clinically important traumatic distress [25].

### 4.5. Coping Mechanisms

The parents of children diagnosed with a congenital heart defect have to cope with a number of stressors that, over time, can lead to mental health problems [17,66]. However, not all parents who face these stressful situations develop mental health symptoms. Thus, parents’ coping strategies may influence their long-term resilience. For example, increased levels of acceptance and positive reinterpretation were associated with fewer symptoms of anxiety, depression and traumatic stress, whereas increased levels of denial and avoidance were associated with more symptoms [25]. A study by Demianczyk et al. showed that the parents of children diagnosed postnatally with a congenital heart defect may apply fewer coping strategies than the parents of children diagnosed prenatally. Prenatal diagnosis gives parents the opportunity to inform themselves about the diagnosis, to evaluate treatment options, and also to prepare for treatment before the baby is born. On the other hand, the parents of children diagnosed postnatally have to adapt to new information much more quickly and have limited time for preparation before surgery and hospitalization [31]. In this context, these parents do not have enough time to prepare and seek support, and are at higher risk for mental health problems. There is thus a need for a medical and psychosocial team to actively promote adaptive coping strategies used by parents.

### 4.6. Attachment

Maternal fetal attachment is the intensity with which a pregnant parent engages in behaviors of care and interaction with their unborn child. In a study by Ruschel et al., they showed that the diagnosis of a cardiac malformation in the intrauterine period increases maternal–fetal attachment [38].

### 4.7. Life Satisfaction and Mental Health/Wellbeing

Recent studies have shown that the nurse-guided mHealth approach can significantly contribute to reducing the emotional distress of parents of children with congenital heart defects. A pilot study conducted by McKechnie et al., demonstrated the feasibility of the urse-guided mHealth program in reducing the emotional distress of parents of children with congenital heart defects through psychosocial support in the perinatal period with the provision of specific information such as diagnosis of congenital heart defect, medical and surgical treatment, disease progression, recovery period, and nutrition [28]. In a study by Bratt et al., they showed that the parents of children prenatally diagnosed with a congenital heart defect showed a lower sense of coherence and a significantly lower satisfaction with life [37].

### 4.8. Adaptative Processes

When a mother is told during pregnancy that her child has a congenital heart defect, the first stage is shock and hope that the diagnosis is wrong. Mothers go through a dynamic process of adapting to the diagnosis which is also strongly linked to the belief that their child can be treated [35].

Parents of children prenatally diagnosed with a congenital heart defect feel overwhelmed by the amount of information they have to process in a short time. Positive interaction with medical staff is an important factor in parents’ psychological well-being and adjustment to the diagnosis. They appreciate the opportunity to ask questions in an attempt to understand the situation and the compassion they received from the medical staff. However, there are also unpleasant situations in which professionals are rushed in providing information and have a negative influence, representing a barrier in the parents’ understanding and acceptance of the diagnosis [67]. After the diagnosis of a congenital heart defect, parents should be encouraged to find coping mechanisms according to their own resources. Studies have shown that support groups have benefited parents of children with various conditions. These peer support groups can provide psychological support in parents’ understanding and acceptance of the diagnosis, with positive therapeutic outcomes [13,68,69]. In order to improve parental support, a multidisciplinary team is needed in maternal–fetal units. Professionals trained in fetal cardiology and psychology are essential in counseling parents whose children have been diagnosed with a congenital heart defect.

### 4.9. Limitations

**Limitations of the evidence included in the review**

This review has several limitations that should be considered. The included studies were heterogeneous in design (mostly qualitative), sample size, and outcome measures, which limits the comparability and generalizability. Many studies used small, non-random samples, and heavily relied on self-reported data, increasing the risk of selection and measurement bias.

The overall risk of bias was moderate in most studies, with only two classified as low risk. Cross-sectional designs dominated, making it difficult to assess long-term psychological outcomes or causal relationships.

Geographic and cultural diversity among studies, while enriching, may have introduced variability due to the differences in healthcare systems and support structures. Moreover, fathers were often underrepresented, limiting insight into paternal experiences.

Finally, few studies evaluated psychological interventions in a standardized way, and potential publication bias cannot be excluded. These limitations highlight the need for larger, longitudinal, and methodologically rigorous studies to better inform psychosocial care for parents following a prenatal CHD diagnosis.

**Limitations of the review process**

Despite following PRISMA guidelines, this review may have some limitations in its methodology. Although multiple databases were searched, the potential for publication bias remains, as studies with null or negative results may be underrepresented. The inclusion of predominantly qualitative studies limited the possibility of meta-analysis and introduced subjectivity in the data synthesis. Additionally, while study selection and quality assessment were performed independently by two reviewers, discrepancies were resolved through discussion rather than statistical validation, which may introduce interpretive bias. The lack of protocol registration also limits transparency and reproducibility.

Although the literature search was conducted using a broad range of multidisciplinary databases (PubMed, Embase, Scopus, Medline, Clarivate, and Science Direct), we acknowledge that psychology- and nursing-specific databases such as PsycINFO and CINAHL were not included. As a result, relevant studies published in specialized journals indexed exclusively in those databases may have been missed. This could potentially introduce selection bias and limit the comprehensiveness of the psychological and nursing perspectives included in the review.

Additionally, inter-rater reliability was not formally assessed during the study selection process. While decisions were made through consensus between reviewers, the absence of a quantified inter-rater reliability index (e.g., Kappa coefficient) is recognized as a methodological limitation.

### 4.10. Implications for Practice, Policy, and Future Research

The findings underscore the need for integrated psychosocial support as part of standard prenatal care for families facing a CHD diagnosis. In clinical practice, early psychological screening, empathetic communication, and tailored counseling should be implemented routinely. At the policy level, standardized guidelines for psychosocial interventions in fetal cardiology settings are warranted. Future research should prioritize longitudinal, multi-center studies with larger, more diverse populations, and rigorously evaluate the effectiveness of specific interventions to guide evidence-based care.

Despite increasing recognition of the psychological burden in this population, no formal guidelines have yet been established for the provision of psychosocial support to parents following a prenatal CHD diagnosis. This gap highlights the urgent need for structured, evidence-based recommendations.

## 5. Conclusions

This synthesis highlights that parents whose children have been prenatally diagnosed with a congenital heart defect are at an increased risk of psychological distress. To improve the support offered to these parents, a multidisciplinary prenatal counseling team is needed to provide expecting parents with the most accurate information regarding the diagnosis, potential outcomes, and available interventions. This multidisciplinary team enhances the quality of care and facilitates a more informed and compassionate journey throughout this period.

## Figures and Tables

**Table 1:** Characteristics of the studies included in the systematic review.

Author, Publication Year	Study Design Ant Type	Location	Number of Parents Expecting Children with CHD Included, Controls, Age	Type of Tools Used for Evaluation (Questionnaires, Interviews, Scales)	Follow-Up Period	Specific Domain of Research Related to Parental Psychological Issues	Main Outcomes	Predictors or Modifiers of Psychological Distress	Recommended Interventions for Reduction in Psychological Distress
Vieira, 2025 [27]	Case–control study; quantitative	Porto Alegre, Brazil	50 puerperal women: 23 mothers with prenatal CHD diagnosis of the fetus age 32.6 ± 5.3 and 27 controls (mothers with postnatal CHD diagnosis of their child) age 27.2 ± 5.9 years	Semi-structured questionnaire, Edinburgh Postnatal Depression Scale	-	Depressive symptoms	Prenatal diagnosis of CHD was associated with significantly lower levels of depressive symptoms (26.1% at prenatally vs. 77.8% at postnatally diagnosis)	Time of diagnosis	Fetal diagnosis should be offered to all mothers
McKechnie, 2023 [28]	Prospective study; qualitative	Minneapolis, Houston, and Madison, USA	19 mothers/birthing persons and 15 caregiving partners, age 33.5 (32–36.5) years	Online surveys, session transcripts, and app use	12 weeks postnatally	Mental health/wellbeing	Regulating emotions and co-parenting consistently needed support		Use nurse–parent collaborative in preparing heart and mind topics
Erbas, 2023 [29]	Longitudinal study; quantitative	Munich, Germany	77 parents (45 women and 32 men), no controls, 33.7± 5.262 years	Hospital Anxiety and Depression Scale questionnaire	5–13 months after the birth of the child	Psychological state (anxiety and depression)	Prevalence for prenatal anxiety was 11.8% and for depressed mood 6.6%	Level of education, health and social workers, first-time mothers and parents whose pregnancies were due to medical assistance	The support of the affected parents can positively impact the treatment of the child and should be integrated into the daily routine of the clinic
Mangin-Heimos, 2022 [30]	Prospective longitudinal study; quantitative	St. Louis, USA	43 mothers, 28.2 (23.4–33.0) years, and 36 partners, 30.6 (25.7–33.3) years, no controls	Depression Anxiety Stress Scales	Prenatal, birth, discharge, post-discharge	Psychological distress	Psychological distress was present in 42% (18/43) of mothers and 22% (8/36) of fathers	Low social support for mothers and a history of mental health conditions for fathers	These data suggest that early and repeated psychological screening is important once a fetal CHD diagnosis is made and that providing mental health and social support to parents may be an important component of their ongoing care
Demianczyk, 2022 [31]	Cross-sectional study; qualitative	Philadelphia and Delaware, USA	34 parents (20 mothers and 14 fathers), no controls	Semi-structured interviews—COPE Inventory	1–3 years postnatally	Coping strategies (adaptive and maladaptive strategies)	Mothers were more likely than fathers to report a focus on and venting of emotions (70% vs. 21.43%) and behavioral disengagement (25% vs. 0%)	Time of diagnosis	Interventions tailored to the needs of mothers and fathers for coping strategies are needed to promote adaptive coping and optimize family psychosocial outcomes
Wu, 2020 [32]	Longitudinal, prospective, case–control study; quantitative	Washington, USA	48 pregnant women carrying fetuses with CHD age 32.7 ± 5.5 years and 92 healthy volunteers with low-risk pregnancies, age 33.7 ± 5.4 years	Perceived Stress Scale, Spielberger State-Trait Anxiety Inventory, and Edinburgh Postnatal Depression Scale	-	Maternal psychological distress, anxiety, and depression	65% of mothers tested positive for stress, 44% for anxiety, and 29% for depression	Fetuses with single-ventricle CHD	Psychological distress among women carrying fetuses with CHDs is prevalent and is associated with impaired fetal cerebellar and hippocampal development; efforts should be made to decrees this distress
Harris, 2020 [33]	Quantitative	Nashville, USA	16 mothers, age 30.0 [27.3–34.8] years), 8 fathers, and 3 support individuals age of family member or support individual, 30.0 [26.0–42.0] years), no controls	Audio recorded telephone interviews	1 prenatal follow-up visit and 1 postnatal follow-up visit	Prenatal experience, particularly aspects they found to be stressful or challenging	Uncertainty was identified as a pervasive central theme and was related both to concrete questions on scheduling, logistics, or next steps, and long-term unknown variables concerning the definitiveness of the diagnosis or overall prognosis		Potential future interventions to improve parental support were identified in the areas of expectation setting before the referral visit, communication in clinic, and identity formation after the new diagnosis
Bratt, 2019 [34]	Prospective study; quantitative	Gothenburg, Boras and Trollhattan, Sweden	8 couples age 31.5± 4.1 years and 152 controls age 30.8 ± 4.7 years (pregnant women with a normal screening ultrasound examination)	Hospital Anxiety and Depression Scale, sense of coherence, life satisfaction, and Dyadic Adjustment Scale	2–6 months after delivery	levels of parental distress	The prenatal diagnosis of CHD led to lower sense of coherence, higher levels of anxiety and lower life satisfaction	Time of diagnosis	Parents with a prenatal diagnosis of CHD should be supported through the pregnancy
Im, 2018 [35]	Cross-sectional study; qualitative	Seoul, Republic of Korea	12 mothers, median age 31.5 years, no controls	In-depth interview	1–6 months	Adaptive processes during pregnancy	Mothers went through a dynamic process of adapting to the unexpected diagnosis of CHD, which was closely linked to being able to believe that their child could be treated	Provision of accurate health advice and emotional support by a multidisciplinary counseling team	Early counseling with precise information on CHD, continuous provision of clear explanations on prognosis, sufficient emotional support, and well-designed prenatal education programs are the keys to an optimal outcome
Carlsson, 2016 [23]	Quantitative	Stockholm and Uppsala, Sweden	26 parents of a fetus with CHD (14 mothers, 12 fathers)	Semi-structured telephone interviews	-	Need for information	Individuals faced with a prenatal diagnosis of a congenital heart defect need individualized and repeated information		Information regarding pregnancy termination is needed
Pinto, 2016 [36]	Prospective cohort study; quantitative	Salt Lake City, USA	60 families with prenatal CHD diagnosis, 45 families with postnatal CHD diagnosis, average age of parents (mothers 28.2 versus 27.6 years, fathers 29.9 versus 29.2 years)	Basic Symptom Inventory	At birth, and follow-up	Psychological stress	Parents of prenatally diagnosed infants with CHD had lower anxiety and stress than those diagnosed postnatally after adjusting for severity; scores for anxiety and stress were primarily lower in fathers	Timing of diagnosis	Fetal diagnosis should be offered to all mothers
Carlsson, 2015 [22]	Qualitative	Stockholm and Uppsala, Sweden	11 parents of a fetus with CHD (6 fathers and 5 mothers)	Semi-structured interviews	-	Parental experiences and need for information following a prenatal diagnosis of CHD	Three different themes emerged: “Grasping the facts today while reflecting on the future”, “Personal contact with medical specialists who give honest and trustworthy information is valued”, and “An overwhelming amount of information on the Internet”		Early and honest information in line with individual preferences is crucial to support the decisional process regarding whether to continue or terminate the pregnancy; the use of illustrations is recommended, as a complement to oral information, as it increases comprehension and satisfaction with obtained information
Bratt, 2015 [37]	Qualitative	Gothenburg, Sweden	6 couples, age 33 (24–37) years	Interviews performed 5–9 weeks after a prenatal diagnosis of congenital heart disease	-	Experiences of counselling and need for support during continued pregnancy following a prenatal diagnosis of a CHD	The analysis resulted in three themes: 1/Counselling and making a decision-the importance of knowledge and understanding; 2/Continued support during pregnancy; 3/Next step—the near future	Web-based information of high-quality, written information, support from parents with similar experiences and continued contact with a specialist liaison nurse	Continued support throughout pregnancy was considered important
Bevilacqua, 2013 [20]	Cross-sectional; quantitative	Rome, Italy	38 couples, 20 with prenatal diagnosis of CHD (mothers age 33.7 ± 5.9 years, fathers age 36.1 ± 6.5 years) and 18 with postnatal diagnosis of CHD (mothers age 32.8 ± 5.2 years, fathers age 36.8 ± 7.1 years)	Three self-administered questionnaires (General Health Questionnaire-30, Beck Depression Inventory—Second Edition, Health Survey-36)	-	Emotional distress, depression, and quality of life	Stress and depression levels were significantly higher in mothers than in fathers (stress: 81.8% mothers versus 60.6% fathers; depression: 45.7% mothers versus 20.0% fathers); mothers receiving prenatal diagnosis were more depressed, whereas those receiving postnatal diagnosis were more stressed; fathers showed same tendency	Sex of the parent	Parents of children diagnosed prenatally may need counseling throughout pregnancy to help them recover from the loss of the imagined healthy child
Ruschel, 2013 [38]	Cohort study; quantitative	Porto Alegre, Brazil	197 pregnant women were included, 96 with a fetus with CHD age 28.97 6.89 years and 101 with a fetus without CHD age 27.61 6.40 years	Validated Maternal–Fetal Attachment Scale	After 30 days	Maternal–fetal attachment	Diagnosis of fetal heart disease increases the level of maternal–fetal attachment	Time of diagnosis	Fetal diagnosis should be offered to all mothers
Rychik, 2012 [25]	Cross-sectional survey; quantitative	Philadelphia, USA	59 mothers having a fetus with CHD, age 30± 7 years	Self-report instruments (Impact of Events Scale-Revised, Beck Depression Index II, State-Trait Anxiety Index, COPE Inventory, Dyadic Adjustment Scale)	-	Maternal stress traumatic stress, depression, and anxiety	Post-traumatic stress (39%), depression (22%), and anxiety (31%) are common after prenatal diagnosis of CHD; lower partner satisfaction was associated with higher depression and higher anxiety	Coping skills, partner satisfaction and demographics	Healthy partner relationships and positive coping mechanisms can act as buffers
Brosig, 2007 [39]	Cross-sectional; quantitative	Wisconsin, USA	10 couples with prenatal CHD diagnosis and 16 couples with postnatal CHD diagnosis	Brief Symptom Inventory, Interview		Psychological distress	The severity of the child’s heart lesion at diagnosis was related to parental distress levels; parents with children with more severe lesions had higher BSI scores	Severity of the child’s heart lesion	Results suggest the need to provide parents with psychological support, regardless of the timing of diagnosis
Sklansky, 2002 [40]	Prospective study; quantitative	San Diego, USA	29 mothers with prenatal CHD diagnosis, 184 mothers with normal fetal echocardiography, 28 mothers with neonatal CHD diagnosis	Questionnaire	After birth in the neonatal period	Maternal psychological impact	When fetal CHD was diagnosed, maternal anxiety typically increased, and mothers commonly felt less happy about being pregnant, less responsible for their infants’ defects and tended to have improved their relationships with the infants’ fathers	Time of diagnosis	Fetal diagnosis should be offered to all mothers; it is a tool with great psychological and medical impact

**Table 2:** ROBINS-I risk of bias assessment of quantitative studies included in the systematic review.

Study	Confounding	Selection	Classification	Deviations	Missing Data	Measurement	Reporting	Overall Risk
Vieira [27]	Moderate	Moderate	Moderate	Low	Moderate	Moderate	Moderate	Moderate
Erbas [29]	Low	Low	Low	Low	Moderate	Moderate	Low	Low
Mangin-Heimos [30]	Low	Low	Low	Low	Moderate	Moderate	Low	Low
Wu [32]	Moderate	Moderate	Low	Low	Moderate	Moderate	Moderate	Moderate
Bratt [34]	High	High	Low	Low	High	High	Moderate	High
Pinto [36]	Moderate	Moderate	Moderate	Low	Moderate	Moderate	Moderate	Moderate
Bevilacqua [20]	High	Moderate	Moderate	Low	Moderate	Moderate	Moderate	Moderate
Ruschel [38]	Moderate	Low	Low	Low	Moderate	Moderate	Low	Moderate
Rychik [25]	High	Moderate	Low	Low	Moderate	High	Moderate	Moderate
Brosig [39]	High	Moderate	Low	Low	Moderate	High	Moderate	Moderate
Sklansky [40]	Moderate	Moderate	Low	Low	Moderate	Moderate	Moderate	Moderate

**Table 3:** CASP (Critical Appraisal Skills Programme) risk of bias assessment of qualitative studies included in the systematic review.

Study	CASP Score (/10)	Quality Level
McKechnie [28]	7	Moderate
Demianczyk [31]	6	Moderate
Harris [33]	5	Low–Moderate
Im [35]	6	Moderate
Carlsson [23]	7	Moderate
Carlsson [22]	6	Moderate
Bratt [37]	5	Low–Moderate

## Data Availability

No new data were created or analyzed in this study. Data sharing is not applicable to this article.

## Supplementary Materials

The following supporting information can be downloaded at https://www.mdpi.com/article/10.3390/children12081095/s1, File S1: Search strategy; File S2: Full-text articles excluded after eligibility assessment; File S3: Classification of included studies by design type.
