# Supplementary material for: Parental Psychological Response to Prenatal Congenital Heart Defect Diagnosis
Source: Children (Basel). 2025 Aug 20;12(8):1095. doi: 10.3390/children12081095 (PMC12384161; doi:10.3390/children12081095)
Supplement: Supplementary file 1 [file children-12-01095-s001.zip › Supplementary File S1.pdf]

Search strategy

This document provides the full search strategies used in the systematic review titled "Psychological impact of prenatal diagnosis of congenital heart defects on parents". The searches were conducted in July 2025 across the following databases: PubMed, Embase, Scopus, Medline, Web of Science, ScienceDirect, and Cochrane Library.

The search strategy included the following terms and combinations:
"congenital heart defect" OR "CHD" AND "prenatal diagnosis" AND "psychological impact" OR "parental distress" OR "coping".

Boolean operators and MeSH terms were used when available. No date or language restrictions were applied. Filters were applied to include only human studies.

PubMed
("congenital heart defect"[MeSH Terms] OR "CHD"[Title/Abstract])
AND "prenatal diagnosis"[MeSH Terms]
AND ("psychological impact"[Title/Abstract] OR "parental distress"[Title/Abstract] OR coping[Title/Abstract])
Filters: Humans, No date restrictions, English and other languages

Embase
('congenital heart defect'/exp OR 'CHD':ab,ti)
AND 'prenatal diagnosis'/exp
AND ('psychological impact':ab,ti OR 'parental distress':ab,ti OR coping:ab,ti)
Filters: Human, all languages

Scopus
TITLE-ABS-KEY ("congenital heart defect" OR CHD)
AND TITLE-ABS-KEY ("prenatal diagnosis")
AND TITLE-ABS-KEY ("psychological impact" OR "parental distress" OR coping)
AND (LIMIT-TO (DOCTYPE, "ar"))

Web of Science
TS=("congenital heart defect" OR CHD)
AND TS=("prenatal diagnosis")
AND TS=("psychological impact" OR "parental distress" OR coping)

ScienceDirect
("congenital heart defect" OR CHD) AND ("prenatal diagnosis") AND ("psychological impact" OR "parental distress" OR coping)
Filters: Research articles only

Cochrane Library
"congenital heart defect" AND "prenatal diagnosis" AND ("psychological impact" OR "parental distress" OR coping)
Filters: Trials, Reviews; Human studies

Medline
("congenital heart defect" OR CHD) AND ("prenatal diagnosis") AND ("psychological impact" OR "parental distress" OR coping)
Filters: Humans
