# Supplementary material for: Parental Psychological Response to Prenatal Congenital Heart Defect Diagnosis
Source: Children (Basel). 2025 Aug 20;12(8):1095. doi: 10.3390/children12081095 (PMC12384161; doi:10.3390/children12081095)
Supplement: Supplementary file 1 [file children-12-01095-s001.zip › Supplementary File S2.pdf]

Full-text articles excluded after eligibility assessment

No.	Study citation (Author, Year)	Reason for exclusion
1	Smith et al., 2015	Population not relevant – study focused on healthcare providers' experiences rather than parents.
2	Kim et al., 2018	Population not relevant – included only neonatal outcomes without reference to parental psychological response.
3	Gonzalez et al., 2017	No psychological outcome measures – focused exclusively on surgical preparation and outcomes.
4	Liao et al., 2016	No psychological outcome measures – addressed communication models without measuring distress or coping.
5	Tanaka et al., 2013	No psychological outcome measures – study concentrated on diagnostic imaging modalities.
6	Ivanov et al., 2012	No psychological outcome measures – evaluated cost-effectiveness of prenatal screening only.
