# Supplementary material for: Parental Psychological Response to Prenatal Congenital Heart Defect Diagnosis
Source: Children (Basel). 2025 Aug 20;12(8):1095. doi: 10.3390/children12081095 (PMC12384161; doi:10.3390/children12081095)
Supplement: Supplementary file 1 [file children-12-01095-s001.zip › Supplementary File S3.pdf]

Classification of included studies by design type

No.	Study (Author, Year)	Design type	Methodological approach
1	Vieira et al., 2025	Quantitative	Case-control study, EPDS
2	McKechnie et al., 2023	Qualitative	Online transcripts, narrative analysis
3	Erbas et al., 2023	Quantitative	Longitudinal study, HADS
4	Mangin-Heimos et al., 2022	Qualitative	Longitudinal distress trajectories (DASS)
5	Demianczyk et al., 2022	Qualitative	Semistructured interviews, COPE
6	Wu et al., 2020	Quantitative	Case-control, STAI, EPDS, PSS
7	Harris et al., 2020	Qualitative	Telephone interviews
8	Bratt et al., 2019	Qualitative	Psychological scales + interviews
9	Im et al., 2018	Qualitative	In-depth interviews
10	Carlsson et al., 2016	Qualitative	Semistructured interviews
11	Pinto et al., 2016	Qualitative	Prospective cohort with interviews
12	Carlsson et al., 2015	Qualitative	Interviews on informational needs
13	Bratt et al., 2015	Qualitative	Parental counseling interviews
14	Bevilacqua et al., 2013	Quantitative	GHQ-30, BDI-II, SF-36
15	Ruschel et al., 2013	Qualitative	Maternal-Fetal Attachment Scale
16	Rychik et al., 2012	Qualitative	IES-R, COPE Inventory, descriptive
17	Brosig et al., 2007	Qualitative	Interviews, Brief Symptom Inventory
18	Sklansky et al., 2002	Quantitative	Questionnaire-based survey
